# *In vivo* collection of follicular fluid and granulosa cells from individual follicles of different diameters in cattle by an adapted ovum pick-up system

**DOI:** 10.1186/1477-7827-11-73

**Published:** 2013-08-02

**Authors:** Eduardo KN Arashiro, Miller P Palhao, Sabine Wohlres-Viana, Luiz GB Siqueira, Luiz SA Camargo, Marc Henry, Joao HM Viana

**Affiliations:** 1 Federal University of Minas Gerais, Belo Horizonte, MG 30123-970, Brazil; 2 University Jose do Rosario Vellano, Alfenas, MG 37130-000, Brazil; 3 Federal University of Juiz de Fora, Juiz de Fora, MG 36036-900, Brazil; 4 Embrapa, Juiz de Fora, MG 36038-330, Brazil

**Keywords:** Follicle aspiration, Follicle biopsy, Bovine

## Abstract

**Background:**

Most studies on granulosa cell (GC) function in cattle have been performed using GC and follicular fluid (FF) samples collected from slaughterhouse ovaries. Using this approach, the follicular developmental stage and functional status are unknown and indirectly inferred, limiting data interpretation. Ultrasound-guided follicle aspiration has previously been used to recover GC or FF samples, but this was mostly carried out in large follicles or pools of small follicles, without recording the efficiency of recovery. The present study was aimed at adapting and evaluating an ovum pick-up (OPU) system for the in vivo recovery of FF and GC from individual follicles of different diameters.

**Methods:**

In the first trial, the losses of fluid inside the tubing system were calculated using a conventional or an adapted-OPU system. Blood plasma volumes equivalent to the amount of FF in follicles of different diameters were aspirated using a conventional OPU Teflon circuit. The OPU system was then adapted by connecting 0.25 mL straws to the circuit. A second trial evaluated the efficiency of FF recovery in vivo. Follicles ranging from 4.0 to 16.8 mm in diameter were aspirated individually using the conventional or adapted-OPU systems. A third trial assessed the in vivo recovery of GC and the subsequent amount of RNA obtained from the follicles of different diameters from Holstein and Gir cattle.

**Results:**

In Trial I, the plasma recovery efficiency was similar (P > 0.05) for the volumes expected for 12 and 10 mm follicles, but decreased (P < 0.05) for smaller follicles (45.7+/−4.0%, 12.4+/−4.3% and 0.0+/−0.0% for 8, 6, and 4 mm follicles, respectively). Using the adaptation, the losses intrinsic to the aspiration system were similar for all follicle diameters. In Trial II, the expected and recovered volumes of FF were correlated (r = 0.89) and the efficiency of recovery was similar among follicles <12 mm, while larger follicles had a progressive increase in FF losses that was not related to the tubing system. In Trial III, the number of GC and amount of RNA obtained were not affected (P > 0.05) by follicle size, but differed according to breed (615,054+/−58,122 vs 458,095+/−36,407 for Holstein and Gir, respectively; P < 0.05).

**Conclusions:**

The adapted-OPU system can be successfully used for the in vivo collection of FF and GC from follicles of different diameters. This will enable further endocrine, cellular, and gene expression analyses.

## Background

The granulosa cell layer is a very important component of the ovarian follicle. Interacting with the oocyte and the other cells of the follicle wall, the granulosa cells (GC) synthesize and secrete local factors associated with the regulation of follicle development [1-3] and are the main source of follicular fluid and plasma estradiol [4]. The role of the GC in follicle development and their relationship with oocyte quality have been the subject of several studies reviewed by [5]. However, the internal position of the ovaries and the small size of the follicles complicate the recovery of GC in vivo. Therefore, their availability for analysis remains limited [6]. To overcome this technical limitation, most studies use follicular fluid (FF) and GC collected from slaughterhouse ovaries [7-11] or after ovariectomy [12-14]. Although less complex and less expensive, these approaches do not consider the dynamics of folliculogenesis. In ovaries recovered from slaughterhouses, the stage of follicle development and functional status must be indirectly estimated by size, appearance, or the ratio of estradiol to progesterone (E2/P4) [15,16]. Additionally, little or no information regarding the reproductive status of the animal or environmental effects are available. Follicular dynamics can be monitored before ovariectomy, but any further information about fertility is lost. If granulosa cells are kept under in vitro culture conditions after being collected, an additional bias may be added due to premature luteinization and its associated steroidogenic pattern changes [17]. Therefore, even though the function of the GC has been widely studied, all these aspects should be considered when interpreting the results of these studies.

The in vivo collection of FF and GC provides a wide range of possibilities for studying ovarian physiology but also involves in some difficulties. The conventional procedures and the equipment used for OPU were designed to recover cumulus-oocyte complexes from most of the follicles at once, but not to aspirate or sample them individually. Although the in vivo recovery of FF and GC using OPU systems have been previously reported, in most cases, the samples were collected from large or preovulatory follicles [18-21], or were pooled [22]. Moreover, the amount of FF or GC recovered from each follicle was seldom reported [22-25], and this is a key piece of information that is required to design endocrine and molecular studies on an individual basis.

Whether OPU systems can be used to effectively sample small and medium follicles is particularly important in studies with Zebu breeds. The diameter of the dominant follicle at deviation [26,27] and at ovulation [28] in *Bos indicus* is smaller than in *Bos taurus*. Thus, studies in Zebu and crossbreed animals would require the sampling follicles of smaller sizes than those previously reported in other breeds.

The aim of the present study was to adapt and evaluate the efficiency of an OPU system in recovering individual samples of FF and GC from follicles of different diameters in vivo.

## Methods

### Experimental design and animals

All experimental procedures performed on the animals were previously approved by the Ethics for Animal Use Committee of the Embrapa Dairy Cattle Research Center (Protocol CEUA-CNPGL n^o^: 02/2011). To adapt and evaluate the further use of an OPU system for the recovery of FF and GC samples from follicles of different diameters in vivo, three experimental trials were performed. The first trial was designed to adapt the system to collect FF and GC samples from follicles of different diameters. The second and third trials were designed to evaluate the efficiency of the adapted OPU system for the in vivo recovery of samples of FF and GC, respectively. Crossbred Holstein-Zebu cows (n = 30) were used in Trial II and Holstein (n = 20) and Gir (n = 10) heifers were used in Trial III. All animals were cycling and no reproductive abnormalities were observed in prior gynecological exams. The crossbred cows were kept in an outdoor grazing system (*Brachiaria decumbens*) and the Holstein heifers were confined in free stalls and fed with corn silage and concentrate. Water and minerals were available to the animals ad libitum.

### Trial I: development of the adapted OPU system

A preliminary in vitro test was performed to calculate fluid losses using a conventional OPU Teflon tubing circuit that was 80 cm long and of 1.0 mm internal diameter (WTA Tecnologia Aplicada, Cravinhos, Brazil). The known volumes of blood plasma that were expected to correspond to the volume of FF in follicles of 12, 10, 8, 6, and 4 mm in diameter were placed in 1.5 mL tubes and were 0.90, 0.52, 0.27, 0.11, and 0.03 mL, respectively. These volumes were determined by the formula, 4/3πr^3^, where r = follicle diameter/2. Five replicates were prepared for each follicle diameter. Thereafter, these volumes were aspirated using a 20 G needle connected to one tip of the Telfon circuit and were recovered in separate 1.5 mL tubes located at the end of the system using a vacuum pressure of 80 mm/Hg. These 1.5 mL tubes were weighed prior to the experiment (initial weight - iw). The blood plasma that was recovered was also weighed (final weight - fw) and the volume was calculated using the equation for density (d = w/v, d - density; w – weight; v - volume). Briefly, v = (iw – fw)/d, and the density of the blood plasma was taken to be 1.06 g/mL.

**Figure 1:**
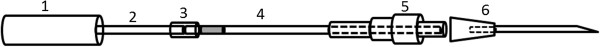
**An illustration of the adapted OPU system.** A 0.25 mL semen straw (4) was placed between the Teflon circuit (2) and the needle (6). To connect the straw, a small silicone tube (3) was used to hold one tip to the circuit, while the other tip was directly connected to the needle (6). For follicles from 7.5 to 8.5 mm in diameter, a second 0.25 mL semen straw was placed between the first straw (4) and the needle (6). The cotton plug of the second straw was removed and another small silicone tube was used to connect the two semen straws. **1**, needle guide; **2**, Teflon circuit; **3**, silicone tube; **4**, 0.25 mL semen straw; **5**, needle adapter; **6**, 20G needle.

The conventional OPU system was later adapted to reduce the losses observed for 8, 6, and 4 mm follicles. A sterile 0.25 mL semen straw was connected between the Teflon circuit and the aspiration needle, so that the FF sample was recovered directly into the straw, without passing through the Teflon circuit (Figure 1). Using this adapted OPU system, known amounts of blood plasma, expected to correspond to the volumes of FF in follicles of 4 and 6 mm in diameter, were aspirated, recovered in the semen straws, and transferred to 1.5 mL tubes. The losses were calculated as previously described. Two 0.25 mL semen straws were connected to aspiration system to recover FF from 8 mm follicles. The cotton plug was removed from the first semen straw.

### Trial II: follicular fluid recovery in vivo using the adapted OPU system

For the second trial, the adapted system (Trial I) was used to collect FF directly from the ovarian follicles of the cows. The emergence of the follicular wave was first synchronized in crossbred cows (n = 30) by the insertion of an intravaginal progesterone releasing device (1 g, Sincrogest, Ourofino Agronegócio, São Paulo, Brazil) and the intramuscular injection of estradiol benzoate (2 mg, Sincrodiol, Ourofino). The animals were scanned daily with a transrectal B-mode ultrasonography (7.5 MHz transducer) to detect the emergence of the new follicular wave. The follicles that were detected in each wave were measured and individually aspirated at specific diameters. They were then grouped into 12 categories, specifically, 4.0 to 4.9, 5.0 to 5.9, and so on, with the last category being composed of follicles > 15 mm.

The main procedures for OPU were carried out as previously described [27]. Follicle aspiration was performed using an ultrasound device (MyLab30 Vet Gold, Esaote, Genova, Italy), equipped with a micro-convex 7.5 MHz transducer connected to a needle guide system (WTA Tecnologia Aplicada). For follicles >8.5 mm, the conventional Teflon tubing system for OPU was used to recover the FF in 1.5 mL tubes. After the aspiration of each follicle, the Teflon system and the needle were replaced to avoid cross-contamination. For follicles ≤7.5 mm, the FF sampling was performed with the adapted system, as previous described. For follicles from 7.5 to 8.5 mm in diameter, two semen straws were used. Instead of a pump, the differential pressure applied to the system to recover the FF was created with a 10 mL syringe. Immediately after follicle collapse, the needle was removed from the ovary and the system was checked for the presence of FF and blood contamination. The volume of the FF sample was determined as previously described (Trial I), based on the sample weight and taking the density of the FF to be 1.03 g/mL.

### Trial III: Granulosa cells recovery using the adapted OPU system

Trial III was aimed at evaluating the number of granulosa cells and subsequent amount of total RNA recovered from follicles of different diameters in vivo in two cattle breeds. The emergence of the follicular wave was synchronized in 20 Holstein and 10 Gir heifers, using the same protocol described in the previous section. The heifers were scanned daily by transrectal B-mode ultrasonography (7.5 MHz transducer) to detect the emergence of the follicular wave. Thereafter, the follicular dynamics were monitored every 12 h and the largest follicle was aspirated at 6, 8, 10 or 12 mm in Holstein, or at 4, 6, 8 or 10 mm in Gir heifers. These diameters were established by taking into consideration the differences in follicle size at deviation between the breeds (8.5 mm for Holstein [29]; 6.5 mm for Gir [27]). The follicle aspiration procedures used were as described in the previous section.

After follicle aspiration, the FF sample volume was measured, as described for Trial II. They were also inspected for the presence or absence of the cumulus-oocyte complex (COC) by stereomicroscope. If present, COC was removed from the fluid. The samples were then centrifuged at 600 X g for 10 min to separate the fluid and the cells. The follicular fluid was removed and stored at −20°C for hormone analysis. The remaining pellet with the GC was re-suspended and vortexed with 200 μL of 0.1% hyaluronidase for 5 min, then washed twice in PBS. The number of cells was determined with a hemocytometer. Aliquots of approximately100,000 cells were then prepared and preserved in RNA later (Ambion, Austin, USA) at −20°C until RNA extraction. The samples that were visually contaminated with blood or with fewer than 100,000 GC were discarded. All statistical analyses only were performed in follicle samples recovered from viable growing follicles, as determined by ultrasound records and the estradiol: progesterone (E2/P4) ratio.

### RNA extraction

Total RNA was extracted from the aliquots of 100,000 cells using the RNeasy Micro Kit (Qiagen GmbH, Hilden, Germany) according to the manufacturer’s instructions and treated with DNase. The total RNA was quantified using 1 μl of sample and a spectrophotometer ND-100 (NanoDrop, Wilmington, USA).

### Hormonal assay

The intrafollicular concentrations of estradiol and progesterone were determined by solid-phase I^125^ radioimmunoassay (RIA), using commercial RIA kits (TKE22 Coat-A-Count Estradiol and TKPG5 Coat-A-Count Progesterone, Siemens Healthcare Diagnostics Inc., Tarrytown, NY, USA), as described previously [30].

### Statistical analyses

The outcome variables, including volume losses, follicular fluid volume, number of GC, and quantity of RNA recovered, were evaluated for normality and homoscedasticity using Lilliefors and Cochran and Bartlett tests. The effects of the follicle diameter and breed were analyzed by ANOVA, and the differences between means among the follicle classes were compared by Tukey’s test. The efficiency of the in vivo recovery of the FF sample was determined by regression analysis. The correlation between follicle diameter and the number of GC recovered in Trial III was compared using Pearson’s correlation method. The frequencies of visible blood contamination and FF samples with less than 100,000 GC were evaluated by Chi-squared test. A probability of P < 0.05 indicated that a difference was significant. The results are shown as the mean ± SEM.

## Results

### Trial I

For the in vitro test, the amount of volume lost to the aspiration system became gradually more significant as the aspirated volume decreased (Table 1). The aspiration of the expected volume for a 4 mm follicle, corresponding to 30 μL, resulted in the complete loss of the fluid to the conventional tubing system. However, when the adapted system was used losses of the expected volumes for follicles of 8, 6, and 4 mm in diameter decreased, and there was no difference (P > 0.05) in losses intrinsic to the aspiration system among follicles of different diameters (Table 1).

**Table 1:** Recovery efficiency in vitro using a conventional (80 cm Teflon tubing) or an adapted (with 0.25 mL semen straws) aspiration system

**Equivalent follicle diameter (mm)**	**Expected FF volume (mL)**	**Recovery efficiency (%)**	
		**Conventional OPU system (n = 5)**	**Adapted system**^**1**^
			**(n = 5)**
12	0.90	87.3 ± 1.1^a^	-
10	0.52	80.1 ± 2.5^a^	-
8	0.27	45.6 ± 4.0^b^	78.3 ± 3.3^a^
6	0.11	12.4 ± 4.3^c^	75.6 ± 4.0^a^
4	0.03	0.0 ± 0.0^d^	73.9 ± 5.0^a^

^a, b, c, d^The means without a common superscript in the same column differed by Tukey’s test (P < 0.05).

^1^ The adapted system was used only for 8, 6, and 4 mm follicles.

### Trial II

**Figure 2:**
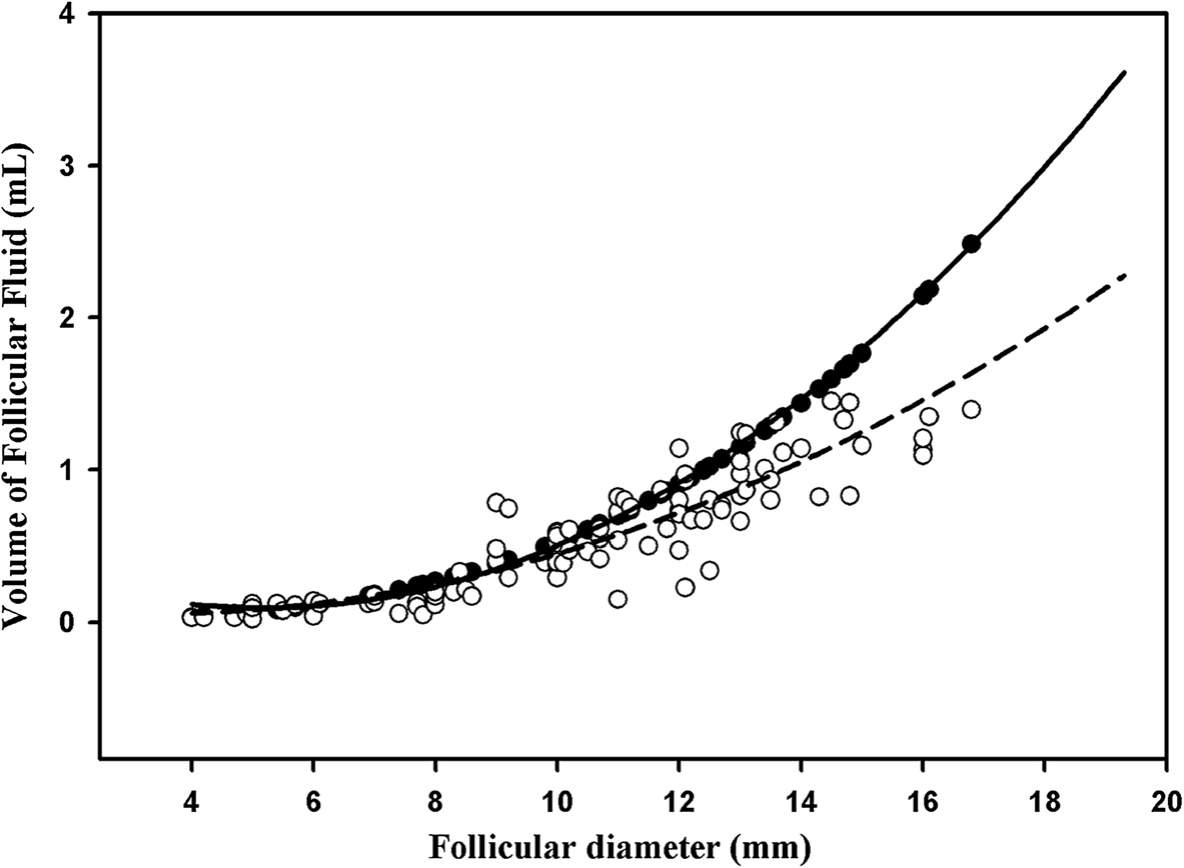
**Follicular fluid recovery according to follicle diameter.** The expected volume (●) and recovered volume (○) of follicular fluid from follicles of different diameters aspirated in vivo using the conventional OPU system (follicles > 8 mm) and the adapted OPU system (follicles ≤ 8 mm). The lines represent the regression curves of the expected ([^**__**^], y = 0.0157x^2^ - 0.1457x + 0.4115; R^2^ = 0.99) and recovered ([---] , y = 0.0069x^2^ - 0.0214x - 0.0258; R^2^ = 0.83) volumes.

Samples of FF were successfully recovered in 96.3% (104/108) of the attempts. There was a high (89.1%, P < 0.05) correlation between the expected and recovered volumes of FF. The volume of FF recovered after aspiration increased with follicular diameter (y = 0.008x^2^-0.05x + 0.14, R^2^ = 0.72). The recovered volume of FF was similar to the expected volumes for follicles with diameter ≤11 mm (P > 0.05). However, in larger follicles, a significant decrease (P < 0.05) in the volume of FF recovered was observed (Figure 2).

### Trial III

The overall mean (±SEM) number of GC recovered was 716,708 ± 68,536, providing 6.8 ± 0.7 samples of 100,000 cells with 14.8 ± 0.7ng of RNA/μL for each punctured follicle. For both breeds follicular diameter had no effect (P > 0.05) on the mean number of GC recovered. However, this was significantly affected by the breed. The overall number of GC recovered was greater in Holstein than in Gir heifers (Table 2). The percentage of FF samples lost due to visible blood contamination was not different between the Holstein and Gir breeds (Table 2). In the Gir breed, visible blood contamination was observed in samples from follicles of 4, 6, 8, and 10 mm (n = 5, 2, 3 and 1 samples, respectively). In the Holstein breed, visible blood contamination was only observed in samples from follicles of 10 and 12 mm (n = 3 and 2 samples, respectively). Similarly, the number of samples that were excluded due to a recovery of fewer than 100,000 GC was not different between the Holstein and Gir breeds (Table 2). In the Holstein group, a low number of GC (<100,000 cells) only occurred in two samples from follicles of 12 mm in diameter, while in the Gir group this was observed in samples from 4 and 8 mm follicles (2 and 1 samples, respectively). The percentage of FF samples excluded due to E2/P4 ratios lower than 1 was the same between the breeds (Table 2). In the Holstein heifers, samples with E2/P4 lower than 1 were observed in samples from follicles of 6 mm in diameter (n = 2) while in Gir heifers, this was observed in samples from follicles of 4 and 8 mm in diameter (n = 2 and 1, respectively). The amount of total RNA extracted from the samples of each follicle was not affected by follicle categories or by breed (8.8 ± 0.7 vs 9.8 ± 1.7 ng/μL, for Holstein and Gir, respectively). No sample was excluded due to failure in RNA extraction.

**Table 2:** Number of GC recovered and the percentage of samples lost due to visible blood contamination, low number of GC (<100,000 cells), or E2/P4 ratio lower than 1

**End Point**	**Hosltein**	**Gir**
*Number of GC recovered*	615,054 ± 58,122^a^	450,095 ± 36,047^b^
*Total RNA extracted (ng/μL)*	8.8 ± 0.7	9.8 ± 1.7
*% of samples lost due to:*		
*Visible blood contamination*	11.4% (5/44)^a^	14.9% (11/77)^a^
*Low number of GC*	4.5% (2/44) ^a^	3.9% (3/77) ^a^
*E2/P4 lower than 1*	4.5% (2/44)^a^	3.9% (3/77)^a^

^a, b^The means without a common superscript in the same column differed by Tukey’s test (P < 0.05).

## Discussion

Most studies in folliculogenesis use follicle fragments, cells or contents recovered from slaughterhouses [7,9,11]. Although practical and less costly, with this approach, little or no information is available about the donor’s reproductive status or environmental effects, thus limiting data interpretation. Additionally, sequential evaluation in the same animal is not possible. The present study evaluated the efficiency of an adapted OPU system for recovering biological materials from individual ovarian follicles in vivo. To our knowledge, this is the first study to describe a methodology for the individual, in vivo aspiration of follicles of different diameters and to quantify the recovered material, specifically, follicular fluid, granulosa cells, and RNA. As expected, it was possible to obtain individual samples of FF, GC, and RNA from follicles larger than 8 mm in diameter using the conventional system. The novelty of the present study was the efficiency of the adapted OPU system in recovering individual samples of FF, GC, and RNA from small antral follicles (4 to 8 mm in diameter). The adaptation (Figure 1) overcame the volume losses observed when the conventional OPU system was used to aspirate volumes from follicles smaller than 10 mm in diameter (Table 1).

The first trial of the present study was aimed at evaluating and adapting the conventional OPU system for FF and GC recovery. FF is composed of colloid proteins and mucopolysaccharides [31] and consequently, shows a variable degree of viscosity [32]. This viscosity causes a speed gradient when fluids pass in tubular systems, with the flux in the inner part of the tube being faster than that in the periphery [33]. Such phenomenon causes the fluids to spread over the inner surface of the aspiration circuits. Consequently, losses of the aspirated volume will be proportionally higher for smaller amounts of fluid, as was observed in the present study. The adaptation performed in the OPU system overcame this problem, making fluid recovery from small (4 mm) follicles viable. When the system was used in vivo (Trial II), the recovery rates were similar in follicles up to 12 mm, but a progressive loss of the expected volume occurred thereafter. This loss was not intrinsic to the aspiration system or to the procedure, because the visualization of large follicles and consequently, their aspiration, was easier. Instead, large follicles are more likely to collapse before complete aspiration, reducing the efficiency of fluid recovery. As such, COC recovery is lower in larger follicles, when compared to smaller ones [34]. In spite of the FF losses in large follicles, the overall efficiency of the technique (recovered/expected) in the present study was high (89.1%). The success rate (samples recovered/follicles punctured) was also greater than those previously reported (96.3% vs 79.2 to 83.3% [24]), demonstrating that the adapted OPU system can be successfully used to obtain FF samples from a range of follicle diameters.

In the second trial, we were able to recover FF samples as small as 28.3 μL (Figure 2). There is a high concentration of steroids, in the FF. For example, estradiol is present in the FF at a concentration of more than 1,000-fold greater than in the plasma [16]. Most FF samples are diluted for steroid analysis [23,35] and therefore, even the small volume recovered from follicles of 4.0 to 5.0 mm is sufficient for evaluating E2 and P4 by RIA, as observed in the present study. For other biochemical analysis, however, the volume of FF may be an important limitation for the individual evaluation of small follicles. For example, in previous studies of the biochemical composition of FF recovered from medium and small follicles, the samples were pooled to reach the necessary volumes [35].

To confirm the status of the follicles aspirated, samples of FF from follicles of different diameters collected in Trial III were evaluated by RIA. The intrafollicular concentration of steroids (E2, P4, E2/P4) can be used as biochemical indices to classify a bovine follicle as healthy or atretic [16]. This classification, however, is retrospective, and cannot be used, for example, to choose which follicles will be sampled. Moreover, despite a general consensus about the use of a E2/P4 ratio >1 as a threshold to classify large follicles as viable or atretic [7,16], there is a huge variation both in estradiol values (26 to 1,776 ng/mL), as well as in the estradiol: progesterone ratio (1.2 to 106) in follicles >8 mm that have been deemed as viable [20]. These differences suggest possible variations in the developmental status or potential of these follicles and suggest that the E2/P4 ratio has limited power as a criterion for selection of follicles for GC studies. In the present study, the adapted OPU system allowed the sampling of FF from follicles after tracking their growth during the follicular wave. Although a few follicles (4.1%; 5/121) still presented E2/P4 ratio < 1, all but one of these were small follicles (<6 mm), in which the expected E2 production is low and an E2/P4 ratio below 1 does not necessarily indicate atresia [15,16]. The total concentration of progesterone (>100 ng/mL) is also used as criteria of atresia [15,36], and when these two criteria were combined, only one follicle in the present study would be classified as atretic. The possibility of tracking follicles before aspiration increases their reliability as a source of biological material for folliculogenesis studies. However, for small follicles, as the period of monitoring is short, there is an increased chance of misidentifying follicles of the same cohort, depending on the resolution of the scanning equipment.

The other possibility evaluated in the present study was the use of the adapted OPU system to recover GC. While the conventional OPU procedure was developed to recover COC [37], mural granulosa cells are also often present in the FF recovered [20]. Interestingly, in the present study, the number of GC recovered did not differ among follicles categories and the correlation between the number of GC cells and FF volume recovered was not significant in both breeds studied (P > 0.05). As the follicle grows, the GC replicates and the number of cells in the follicular wall increases. However, samples obtained from larger follicles did not have more cells than samples obtained from smaller ones. Previous studies suggested that the rates of GC proliferation and of antrum expansion are not coordinately regulated [38,39] and that differences between them could influence the number of GC layers as well as their accommodation over the basal membrane [40]. This would indirectly affect the probability of GC being sloughed off during follicle aspiration. Moreover, in small follicles, the tip of the aspiration needle is closer to most of the inner antrum surface and as such, losses of GC together with FF are less likely to occur. This could explain the lack of difference observed in the number of GC recovered from follicles of different diameters.

Blood contamination in FF samples often results in the samples being unusable for GC gene expression analysis. This is because the contamination may lead to a miscount of the cell concentration and interfere with the efficiency of total RNA extraction using the commercial kit. Despite working with a live animal model, in the present study, the number of FF samples lost due to visible blood contamination was low (11.4% and 13.9% for Holstein and Gir, respectively). When the targeted follicle is not successfully aspirated in the first attempt due to the movement of the animal or to difficulties in reaching the follicle antrum, the eventual injury caused by the needle movement increases the likelihood of blood contamination. This limits the number of follicles collected in each procedure. Visible blood contamination occurred in samples recovered from all follicular categories, indicating that the risk of sample loss due to visible blood contamination was not related to follicle diameter. Because of their reduced dimensions, small follicles (≤ 6 mm) are more difficult to aspirate and injuries to the ovarian stroma are more likely to occur. On the other hand, while the aspiration of larger follicles is easier to perform, the follicular wall has a well-developed vascular bed [41]. Consequently, a small contamination by blood cells is likely to occur when aspirating follicles from live donors. The results of the present experiment, however, suggest that visible blood contamination was an infrequent and accidental event that occurs regardless of follicle size.

The amount of total RNA extracted from the samples was unaffected by follicular diameter or breed. In this study, and as has been previously reported, in the GC layer, the cells were strongly adhered to each other and after follicular aspiration and large clusters of GC were observed [20]. Although the GC samples were vortexed with 0.1% hyaluronidase solution, some GC clusters still persisted. The presence of these clusters may have led to a miscount of the GC recovered and consequently, the GC may not have been evenly distributed in the aliquots. A previous study that collected FF and GC samples from preovulatory follicles reported that 30% of the samples collected did not provide enough mRNA for analysis by RT-PCR [21]. The contamination of GC samples with other cell types, such as epithelial cells or fibroblasts, can also occur during follicle aspiration. However, the amount of such contaminants is unlikely to be significant and these cell types would not interfere in the analysis of the expression of the genes particular of interest in GC, especially those in the steroidogenic pathway. Despite these limitations, in the present study most of the FF samples collected in both breeds provided sufficient quantities of GC and quantity of RNA to perform further analysis by PCR.

## Conclusions

The adapted OPU system can be successfully used in vivo to collect samples of FF and GC from follicles of different diameters for further endocrine, cellular, and gene expression analyses.

## Competing interests

The authors declare that they have no competing interests.

## Authors’ contributions

JHMV conceived the project and coordinated the study. JHMV, EKNA, LGBS, and MPP contributed to the experimental activities, including the in vitro trial, animal synchronization, in vivo OPU procedures, and processing of samples. EKNA and MPP performed the hormonal assay. SWV preformed the RNA extraction, reverse transcription, and PCR tests. LSAC and MH contributed to the data and statistical analysis. JHMV and EKNA drafted the manuscript, which was revised by all authors. All authors read and approved the final manuscript.

## Authors’ information

JHMV, LSAC, and LGBS are researchers at the Laboratory of Animal Reproduction of Embapa Dairy Cattle Research Center. MPP is a professor at Universidade José do Rosário Vellano (Unifenas). SWV is a DSc student (Biological Sciences) at Federal University of Juiz de Fora. During the experimental period, EKNA was a DSc student (Animal Science) at Federal University of Minas Gerais and currently, he has a post-doctoral scholarship at Embrapa Dairy Cattle Research Center. MH is a professor at Federal University of Minas Gerais.
